# The TCR and LCK: foundations for T-cell activation and therapeutic innovation

**DOI:** 10.3389/fimmu.2025.1737013

**Published:** 2026-01-12

**Authors:** Nadine M. Woessner, Valeria Uleri, Ondrej Stepanek, Susana Minguet

**Affiliations:** 1 Signaling Research Centers BIOSS and CIBSS, University of Freiburg, Freiburg, Germany; 2 Department of Synthetic Immunology, Faculty of Biology, University of Freiburg, Freiburg, Germany; 3 Laboratory of Adaptive Immunity, Institute of Molecular Genetics of the Czech Academy of Sciences, Prague, Czechia; 4 Faculty of Science, Charles University in Prague, Prague, Czechia; 5 Centre for Chronic Immunodeficiency (CCI), Faculty of Medicine, University of Freiburg, Freiburg, Germany

**Keywords:** immunotherapy, Lck, signaling, T cells, TCR - T cell receptor

## Abstract

The T cell receptor (TCR)-CD3 complex is crucial to adaptive immunity, driving antigen recognition and intracellular signaling cascades. CD3 subunits harbor key cytoplasmic motifs that recruit signaling proteins like LCK. While distal αβ TCR signaling is well-understood, gaps persist in our understanding of proximal signaling, particularly the roles of free *versus* co-receptor CD4 or CD8-associated LCK and their impact on antigen sensitivity and activation thresholds. In contrast to αβ T cells, γδ T cells recognize diverse antigens, often independently of MHC or MHC-like molecules. Despite their shared CD3 signaling components, the proximal signaling mechanisms of γδ T cells remain poorly characterized, raising important questions about their activation pathways and kinase dependencies. Addressing these gaps is essential to unlock the unique therapeutic potential of γδ T cells in cancer immunotherapy. Recent advances in engineered T-cell therapies demonstrate how proximal TCR signaling can be leveraged for therapeutic innovation. Chimeric antigen receptor (CAR) and chimeric-TCR designs that incorporate specific CD3 signaling motifs have shown improved anti-tumor activity, reduced exhaustion, and enhanced persistence, reflecting a shift beyond traditional ζ chain-dominated designs. In parallel, emerging small-molecule modulators targeting early TCR events offer new strategies to tune pathogenic T-cell responses in autoimmunity or to reset exhausted CAR T cells. This review explores the critical roles of CD3 motifs and LCK in TCR activation, with a focus on the underexplored γδ T cells. We also discuss how these insights could drive next-generation cancer immunotherapies and novel treatments for autoimmune diseases and immunopathologies.

## Introduction

TCR signaling is fundamental to adaptive immunity, enabling precise recognition of antigens and the initiation of intracellular cascades that drive T-cell activation. This process is critical for mediating immune responses against infected or malignant cells, but also for the formation of self-tolerant and self-MHC-restricted T-cell repertoire by negative and positive selection of thymocytes. The TCR complex consists of ligand-binding αβ or γδ TCR heterodimers and the signal-transducing chains CD3γ, CD3δ, CD3ϵ, and ζ (also known as TCRζ, CD247 or CD3ζ), collectively known as the CD3 complex. The CD3 cytoplasmic tails harbor key motifs – immunoreceptor tyrosine-based activation motifs (ITAMs), basic-rich stretches (BRS), proline-rich sequences (PRS), and the receptor kinase (RK) motif – that coordinate the recruitment of kinases, scaffolds, and adaptors essential for signal transduction and amplification.

While distal signaling cascades downstream of the αβ TCR are well characterized, our understanding of how proximal signaling events are regulated remains incomplete. Much of what we know comes from studies using cell lines or transgenic and knockout (KO) mice. Although these models have been invaluable, mutations in the TCR components often disrupt TCR assembly or expression, potentially skewing the interpretation of the findings. Recent advances in chimeric receptor technology now allow for the functional testing of specific CD3 signaling motifs in isolation, offering a novel tool to dissect the mechanics of T-cell activation (1–4).

The very first biochemical step in the TCR signaling cascade is the phosphorylation of the ITAMs within the CD3 cytoplasmic tails by the lymphocyte-specific protein tyrosine kinase (LCK), creating docking sites for molecules like the ζ chain-associated protein kinase 70 (ZAP70) that facilitate downstream signal transduction. LCK is a SRC family tyrosine kinase (SFK) with a modular architecture comprising an N-terminal unique domain (UD), a SH3 and SH2 domain, a tyrosine kinase domain (KD), and a C-terminal negative regulatory domain (5, 6). The N−terminal membrane−anchoring domain targets LCK to the inner leaflet of the plasma membrane and allows association with CD4/CD8 coreceptors. The SH3 and SH2 domains mediate intramolecular interactions that maintains LCK in a closed, inactive conformation, whereas phosphorylation of Y394 in the activation loop of the KD drives its full enzymatic activation. Despite significant progress in understanding LCK’s role in αβ T-cell activation, key questions remain about how distinct pools of LCK – free *versus* co-receptor-associated – modulate signaling efficiency, antigen sensitivity, and activation thresholds. Deciphering these mechanisms is essential for unraveling the nuances of TCR signal initiation and propagation.

αβ and γδ TCRs differ significantly in their antigen recognition even though they share signaling subunits and motifs. The αβ TCR interacts with peptide-antigens presented by major histocompatibility complex (MHC) molecules together with the co-receptors CD4 or CD8. In contrast, γδ TCRs recognize a broader spectrum of antigens, including non-peptide ligands such as phosphoantigens, lipids, and stress-induced self-molecules, often independently of MHC or MHC-like presentation (7). These fundamentally different ligands suggest that γδ TCRs may employ unique proximal signal mechanisms, a topic that remains largely unexplored. For decades, the αβ TCR has been considered the canonical model for TCR signaling, with little attention paid to whether γδ-TCR signaling diverges fundamentally. Understanding these distinctions is increasingly important given the emerging potential of γδ T cells in cancer immunotherapy.

Importantly, insights into proximal TCR signaling are no longer of purely mechanistic interest, but increasingly inform therapeutic innovation. Chimeric antigen receptors (CARs) have advanced significantly by incorporating diverse CD3 signaling motifs, optimizing persistence, reducing exhaustion, and enhancing anti-tumor activity of CAR T cells (1–3, 8). Similarly, chimeric TCRs that utilize the full repertoire of CD3 motifs offer new avenues to fine-tune immune responses (4). At the same time, pharmacologic agents that modulate early TCR signaling steps are emerging as precision immunotherapies. Examples include small-molecule inhibitors targeting the PRS-NCK interaction (such as AX-024) (9), the LCK-RK interaction (such as C10) (10), and kinase inhibitors (such as dasatinib), which can reversibly suppress CAR signaling to mitigate cytokine release syndrome (CRS) and restore exhausted cells (11–13). Together, these strategies highlight the therapeutic potential of fine-tuning proximal TCR signaling in autoimmunity, GVHD, and cancer.

In this review, we will explore the roles of CD3 signaling motifs and LCK recruitment in TCR activation, with a focus on their distinct contributions to αβ and γδ T-cell signaling. By challenging the assumption of uniformity across TCR subtypes, we aim to highlight the diversity of signaling mechanisms. Furthermore, we will examine how this knowledge can be applied to improve cancer immunotherapy and to develop targeted therapies for autoimmune diseases and other immunopathologies.

## Structure of the αβ TCR-CD3 complex

**Figure 1:**
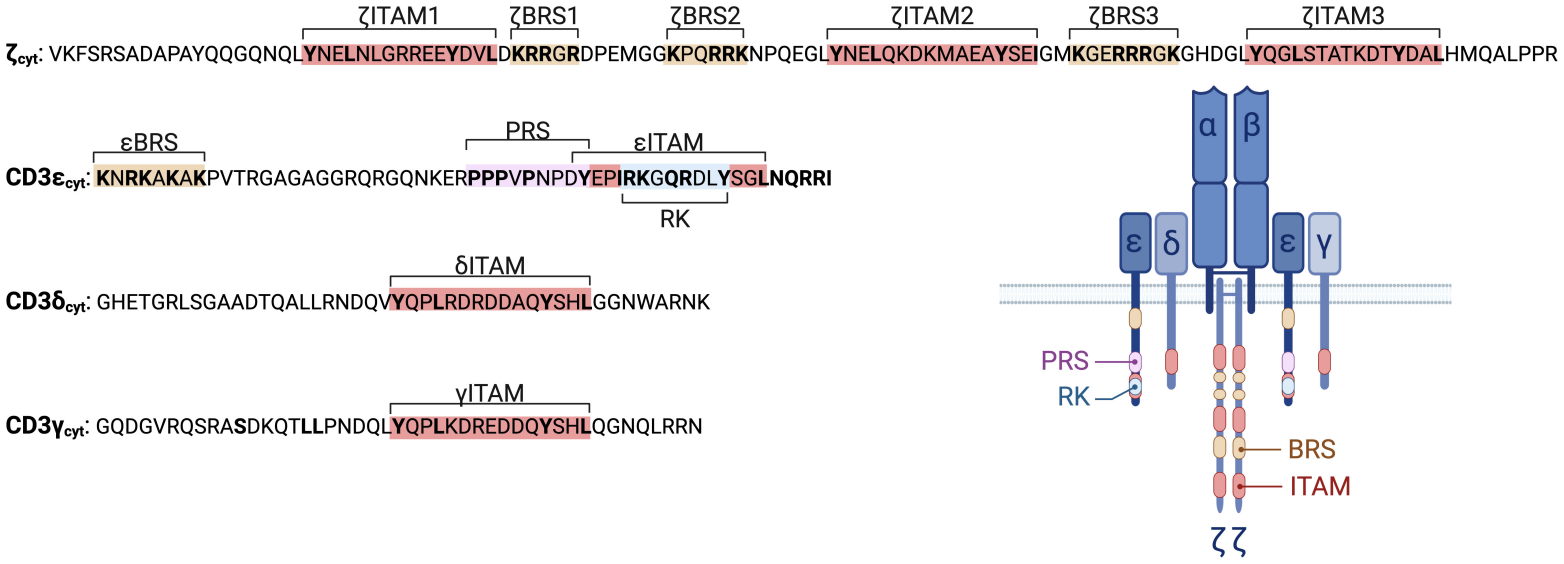
Key signaling motif of the TCR-CD3 complex. The sequences of the human CD3 cytoplasmic tails are shown, highlighting their signaling motifs. In addition, a detailed schematic of the TCR-CD3 complex, showing the αβ TCR chains and the associated CD3 subunits (CD3ϵ, CD3δ, CD3γ, and ζ (also known as TCRζ, CD247 or CD3ζ)) is depicted including the signaling motifs ITAM (red), BRS (yellow), PRS (purple), RK (blue), and their relative positions.

The αβ TCR is a heterodimeric protein complex composed of a single α- and a single β-chain, each containing a constant and variable domain. Together, the αβ heterodimer forms the binding site that recognizes peptide-antigens presented by MHC class I or II molecules (pMHC). The αβ TCR lacks intrinsic signaling capacity. In order to be expressed at the plasma membrane and to signal, the αβ TCR pairs with the CD3 complex, which includes two CD3ϵ, one CD3γ, one CD3δ chain, and a ζζ dimer (**Figure 1**). The CD3 subunits are equipped with distinct cytoplasmic signaling motifs, including ITAMs, BRSs, the PRS, and the RK motif (**Figure 1**). These motifs orchestrate the recruitment of tyrosine kinases LCK and ZAP70 and the adaptor protein NCK, facilitating the initiation and propagation of intracellular signaling cascades. The following sections will explore the unique features of these motifs and their role in αβ TCR signal transduction.

## Key motifs of the CD3 complex

### Immunoreceptor tyrosine-based activation motifs

Immunoreceptor tyrosine-based activation motifs (ITAMs) are crucial for TCR signal transduction. Each ITAM consists of a conserved YxxL/I-X6-8-YxxL/I motif, where the tyrosine residues play a central role in signal initiation (14–16). These tyrosines are phosphorylated upon TCR engagement, predominantly by LCK, creating docking sites for signaling molecules such as ZAP70 (**Table 1**). This phosphorylation is essential, as substituting tyrosine (Y) with phenylalanine (F), a residue that cannot be phosphorylated, renders the ITAM non-functional, effectively abolishing downstream signaling (17). Furthermore, ITAM phosphorylation must be reversible to allow dynamic regulation of TCR signaling. This has been demonstrated using phosphomimetic variants, where glutamic acid substitutions mimic the negative charge of phosphorylated tyrosines, impairing T-cell activation and highlighting the importance of dynamic phosphorylation (18).

**Table 1:** Interaction partners of the distinct signaling motifs as described for the αβ TCR-CD3 complex.

CD3 chain	CD3 motif	Known interaction partners in αβ T cells
ζ, CD3ϵ, CD3γ, CD3δ	ITAM double phosphorylated	ZAP70(SH2) (ζ1, γ, δ > ζ2, ζ3, ϵ), LCK(SH2)
ζ	ζITAMs monophosphorylated	SHP1
ζ	ζBRS	Acidic phospholipids
CD3δ	δITAM monophosphorylated Y1	SHP1
CD3ϵ	ϵITAM monophosphorylated Y1	LCK, CSK
CD3ϵ	ϵITAM monophosphorylated Y2	NCK(SH2)
CD3ϵ	ϵBRS	Acidic phospholipids, GRK2, CAST, LCK(UD), p85(SH3)
CD3ϵ	PRS	NCK(SH3.1), NUMB, EPS8(SH3), p85(SH3)
CD3ϵ	RK	LCK(SH3)
CD3γ	di-Leu	PKC

The TCR-CD3 complex contains ten ITAMs, with one ITAM on the cytoplasmic tails of CD3ϵ, CD3δ, and CD3γ, and three ITAMs on each ζ chain (14–16). ITAMs are not unique to the TCR; various receptors on hematopoietic cells, including the B-cell receptor (BCR), several Fc receptors, and certain NK-cell receptors signal through cytoplasmic ITAMs (14, 19). Interestingly, receptors that bind abundant or polyvalent antigens, such as the BCR, typically contain two to four ITAMs (20). In contrast, the αβ TCR, which demonstrates extremely high antigen sensitivity by showing transient calcium signaling to even a single agonist pMHC ligand (21, 22), contains a much larger number of ITAMs. This might suggest that the presence of multiple ITAMs in the TCR-CD3 complex contribute to signal amplification. Indeed, studies in murine models have shown that reducing the number of ITAMs below seven per TCR-CD3 complex impairs TCR function during central tolerance in the thymus, leading to autoimmune disorders (23). However, recent findings suggest that modified TCR-CD3 complexes with only four functional ITAMs are more responsive to weak antigens than their wild-type (WT) counterparts with ten ITAMs, reflecting the nuanced roles of ITAMs in modulating signal strength (24).

Moreover, the exact amino acid sequences of each ITAM within the TCR-CD3 complex differ (**Figure 1**), resulting in different phosphorylation efficiency by kinases such as LCK and in varied binding affinities to downstream signaling molecules. This diversity renders ITAMs functionally non-equivalent, with distinct qualitative contributions to TCR signaling (23, 25). Evidence for this functional diversity comes from studies in mice engineered to have a single ITAM sequence across all ten positions. These mice exhibit impaired thymocyte development, failing to transition effectively from double-negative (DN) to double-positive (DP) stages, due to reduced TCR expression and signaling (25).

**Figure 2:**
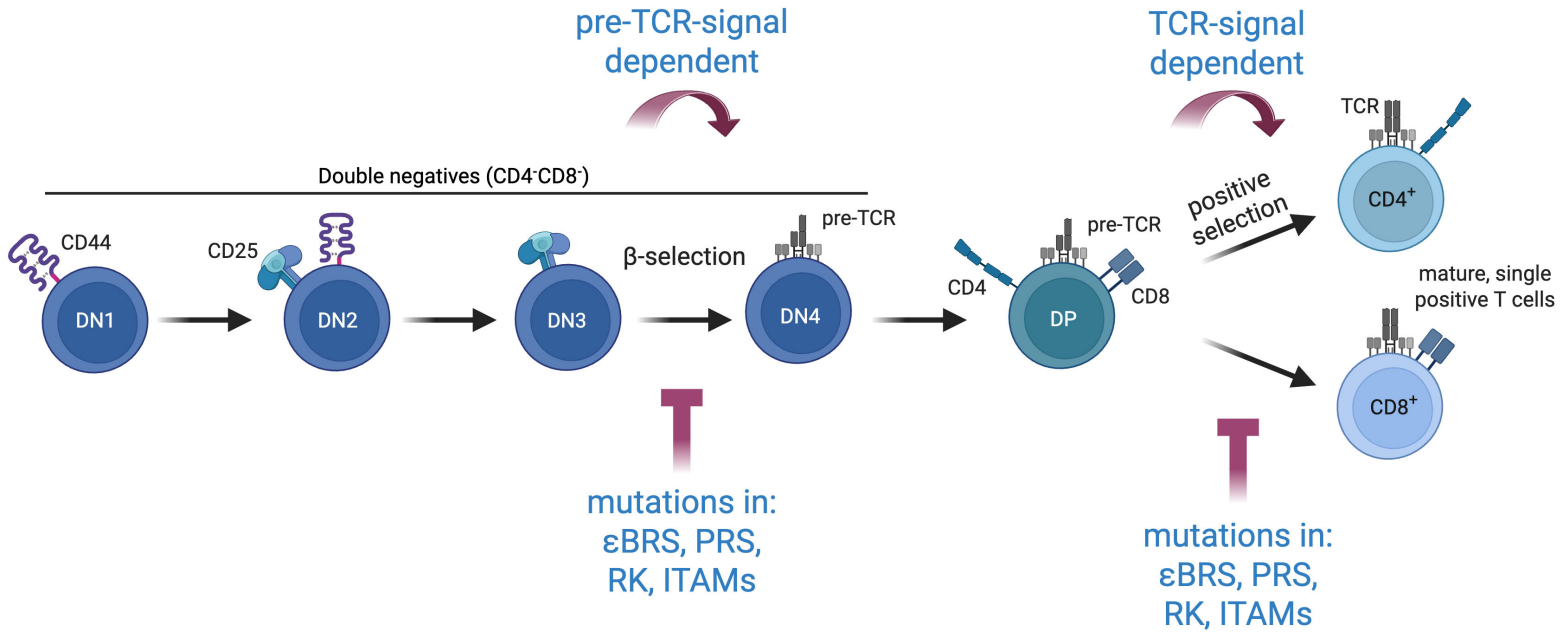
Mutations in key signaling motif affect αβ thymocyte development at check-points where pre-TCR and TCR signaling are required. Loss-of-function mutations of the BRS, PRS or RK motif of CD3ϵ lead to a partial arrest at the DN3-DN4 and the DP-SP transition (2, 38, 45, 46). Similarly, engineering a single ITAM sequence (from either ϵ, δ, γ, ζ1, ζ2 or ζ3) across all ten ITAM positions leads to limited DN to DP transition in thymocytes (25).

Interestingly, ITAMs are not limited to signal activation; some also play regulatory roles. For instance, monophosphorylation of the CD3ϵ ITAM (ϵITAM) recruits the C-terminal Src kinase (CSK) (1), while monophosphorylated ITAMs of CD3δ (δITAM) and ζ (ζITAM) recruit the SH2-containing protein tyrosine phosphatase-1 (SHP1) (3, 24) (**Table 1**). Both are negative regulators of TCR signaling, highlighting the dual role of ITAM phosphorylation in balancing activation with feedback inhibition to regulate T-cell activation. Although our understanding of the qualitative and quantitative roles of individual ITAMs within the TCR-CD3 complex remains incomplete, current data emphasize the importance of ITAM multiplicity and diversity during T-cell development, signaling and tolerance (**Figure 2**).

The unique properties of individual ITAMs are likely influenced by their interaction with other motifs within the CD3 cytoplasmic tails. However, the lack of structural data limits our understanding of these interactions. Cryo-electron microscopy (Cryo-EM) studies of the TCR-CD3 complex have yet to resolve the cytoplasmic tails, due to their high flexibility. This flexibility may facilitate a range of molecular interactions but could also constrain or modulate specific signaling events. Further research into the three-dimensional organization and dynamics of these tails is essential to elucidate how their configurations shape TCR signaling.

### Basic rich stretch

Basic-rich stretch (BRS) motifs are arginine and lysine-enriched amino-acid sequences that mediate electrostatic interactions with nearby negatively charged molecules, such as acidic lipids (26). These motifs are present in several transmembrane proteins, including FcϵRIγ, CD43, CD44, CD28, 4-1BB (CD137) and ICAM1/2 (27), as well as cytosolic proteins (28, 29). Within the TCR-CD3 complex, BRS motifs are found in the cytoplasmic tails of CD3ϵ and ζ (**Figure 1**).

The BRS of CD3ϵ (ϵBRS) is located in the juxtamembrane region of its cytoplasmic tail (30, 31) (**Figure 1**). This motif binds to different acidic phospholipids found in the plasma membrane and various cellular organelles, anchoring CD3ϵ to the membrane (31). The ζ chain, in contrast, contains three BRS motifs: two between ζITAM1 and ζITAM2, and one between ζITAM2 and ζITAM3 (ζBRS1-3) (32).

Landmark studies propose that BRS motifs sequester and protect ITAMs from phosphorylation in the absence of TCR-ligand engagement by binding to phospholipids in the inner leaflet of the plasma membrane (33–36). However, contrary to this protective role, mutations that disrupt the ability of either the εBRS or ζBRSs to interact with the plasma membrane unexpectedly lead to reduced TCR signaling (31, 37). This apparent paradox highlights the complexity of BRS functions. Mice lacking a functional ϵBRS exhibit impaired DN3-DN4 thymocyte transition and defective positive selection, underscoring the importance of the ϵBRS in TCR signaling during T-cell development (38) (**Figure 2**).

Beyond lipid interactions, the ϵBRS has also been reported to interact with signaling proteins, such as GRK2, CAST, LCK and p85 (1, 30, 39, 40) (**Table 1**). However, no protein-protein interactions involving the ζBRSs have been identified to date.

The mechanisms underlying BRS functions remain incompletely understood. Whether their role in TCR signaling depends primarily on lipid binding, protein interactions, or a combination of both is still unclear. Loss-of-function mutations in the ϵBRS, for example, reduce TCR surface levels in both developing and peripheral T cells and impair TCR localization to the immunological synapse (31). These findings suggest a role for the ϵBRS in synapse formation, endosomal recycling, and TCR degradation. However, the precise molecular mechanisms governing these processes require further investigation.

### Proline rich sequence

The TCR contains a Proline rich sequence (PRS) within the CD3ϵ cytoplasmic tail (**Figure 1**), which recruits the adaptor protein NCK through its first Src-homology 3 (SH3.1) domain, facilitating efficient TCR signaling (41) (**Table 1**). This interaction also stabilizes the association between LCK and the TCR, further enhancing signal propagation (42). Additionally, the PRS serves as a binding site for other signaling molecules, including NUMB (43) and EPS8 (44), underscoring its multifunctional role in TCR signaling (**Table 1**).

Studies in PRS-knock-in mice (PxxP to AxxA) revealed significant impairments in T-cell development. Thymocytes exhibited a partial arrest at the DN3-DN4 transition, reduced efficiency in both positive and negative selection at the DP stage, and defects in maturation into CD4 single positive (SP) and CD8 SP thymocytes. All these observations support a role for the PRS at check points where effective pre-TCR and TCR signaling is required (45, 46) (**Figure 2**). Interesting, immature thymocytes in these mice displayed increased TCR surface levels, likely as a compensatory mechanism to the reduced signaling output per TCR molecule.

Furthermore, it was demonstrated that PRS mutations in CD3ϵ selectively affect *in vitro* T-cell responses to weak but not strong antigenic peptides (47). This finding aligns with studies showing that the absence of NCK impairs T-cell activation under weak antigenic stimulation (48). Together, these results highlight the PRS as a key regulator of TCR signaling, particularly in modulating responses to antigens of low strength.

### Receptor kinase motif

A unique feature of the TCR is the receptor kinase (RK) motif, located within the ITAM of the CD3ϵ cytoplasmic tail (**Figure 1**). This motif, defined by the sequence RKxQRxxY, directly recruits the kinase LCK by interacting with its SH3 domain (2) (**Table 1**). Structural studies revealed that the RK motif binds to the RT loop and n-SRC loop of LCK’s SH3 domain. Notably, most RT loop residues are not conserved in related kinases, providing a potential explanation for the unique role of LCK in T-cell development and activation (49, 50).

The specificity of the SH3(LCK)-RK(CD3ϵ) interaction is underscored by the rarity of similar motifs. To date, only two similar motifs have been identified: one in the adaptor protein SKAP1 (RKxx(Y)xxY) (51) and another in *Candida albicans* (52). These findings highlight the exceptional role of the RK motif in mediating TCR-specific recruitment of LCK. Functional studies demonstrate that mutations of the RK motif reduce TCR signaling and T-cell activation and negatively affect thymocyte development (2) (**Figure 2**). These results establish the RK motif as a critical regulator of TCR signaling and highlight its essential role in coordinating proximal T-cell activation.

### Additional motifs

The cytoplasmic tail of CD3ε contains an endoplasmic reticulum (ER) retention motif (NQRRI) that plays a crucial role in regulating the assembly and surface levels of the TCR (53). This motif ensures that only fully assembled TCR-CD3 complexes are transported to the plasma membrane. Mutations in the ER retention sequence allow the surface expression of incomplete αβγϵ and αβδϵ complexes that lack the ζ chains, highlighting its critical quality-control function (54).

CD3γ holds a membrane proximal di-leucine motif involved in TCR downregulation after phosphorylation of the serine residue five amino acids prior by protein kinase C (PKC) (55, 56). This motif has been shown to be important for controlling T-cell homeostasis and the response to virus infections (57, 58). CD3δ also contains a di-leucine motif, which is however missing the precedent serine residue and is therefore currently being considered nonfunctional.

The diversity of motifs within the CD3 subunits highlights their specialized contributions to the TCR function. CD3ϵ stands out as being particularly enriched in signaling and regulatory motifs – including the BRS, PRS, RK, ITAM, and ER retention motif – suggesting a central role in both assembly and signal integration. In contrast, ζ, CD3γ and CD3δ seem to exhibit more singular functions: signal amplification (ζ), downregulating the TCR through the di-leucine motif (CD3γ) or by recruiting the negative regulator SHP1 (CD3δ and ζ). The reasons behind this asymmetry in motif distribution remain an open question. It is possible that evolutionary pressures have tailored CD3ϵ to act as a multifunctional hub, while ζ, CD3γ and CD3δ play more ancillary roles. Future studies focusing on the distinct contributions of these subunits may reveal new layers of complexity in TCR regulation.

## Mechanisms of αβ TCR signal transduction

The precise mechanisms by which the TCR transmits signals upon ligand-engagement remain an area of active investigation and debate. Several models haven been proposed to explain this critical process (reviewed in (59–61)). Some models suggest intrinsic mechanisms within the TCR itself, such as conformational changes, mechanical force, or aggregation, as drivers of signaling. Others portray the TCR as a passive receptor, with activation controlled by tipping the balance between kinases and phosphatases near the engaged receptor, a concept known as kinetic segregation.

**Figure 3:**
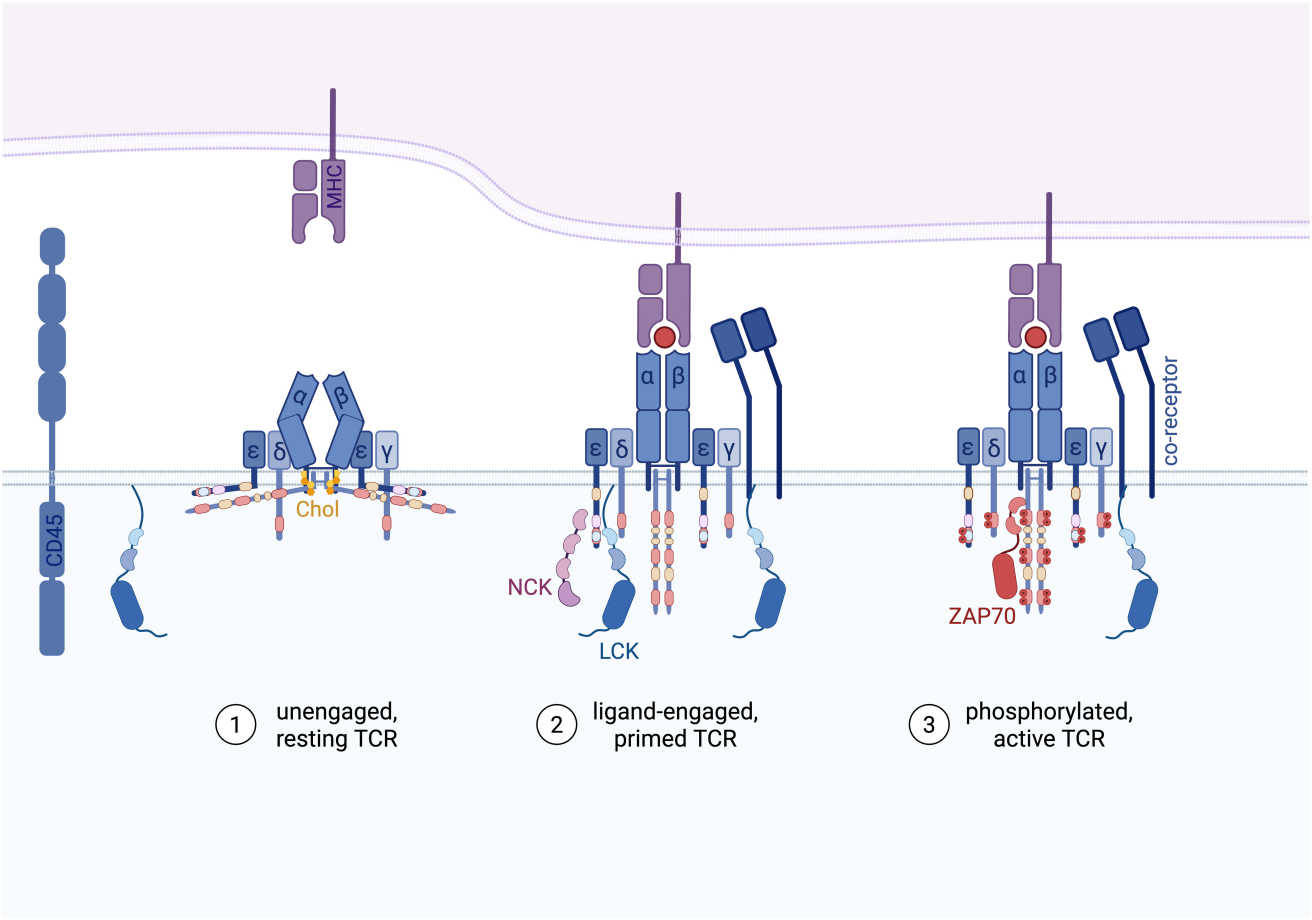
Step-wise proposed model for TCR activation. 1. Unengaged and resting TCR in a closed and compacted conformation. The cytoplasmic tails of CD3ϵ and ζ are sequestered in the inner leaflet of the membrane through the BRS motifs and cholesterol (Chol) locks the TCR in an inactive conformation. CD45 freely diffuses in the membrane, dephosphorylating and preventing spurious activation. 2. Ligand-bound, un-phosphorylated TCR in an open conformation (primed TCR), the CD3 signaling motifs are exposed to recruit the adaptor protein NCK to the PRS motif (purple) as well as LCK to the RK motif (blue) and the BRS (yellow) of CD3ϵ. 3. Active, phosphorylated TCR recruits ZAP70 to the double-phosphorylated ITAMs (red) to mediate downstream signaling pathways. At which state the co-receptors CD8 or CD4, which are associated to LCK, are recruited to the TCR-pMHC complex is still unclear (see below).

As with many other transmembrane receptors, cumulative evidence supports that the TCR functions as an allosteric receptor, cycling between various conformational states that dictate whether signaling cascades are initiated (62, 63). Upon binding to pMHC, the αβ TCR-CD3 complex transitions from its resting, unengaged state into an active state (ligand-engaged TCR; **Figure 3**). This engagement induces further conformational shifts, allowing the receptor to adopt phosphorylated states that were inaccessible before ligand binding. These sequential changes enable the TCR to initiate and propagate intracellular signaling with remarkable specificity and sensitivity.

### Un-engaged TCR

In the absence of ligand binding, the αβ TCR-CD3 complex exists in a compact, resting conformation (**Figure 3**). Cryo-EM studies using nanodiscs aiming to mimic native lipid environments reveal that the TCRαβ ectodomains are tightly associated with the CD3 ectodomains and the transmembrane and juxtamembrane regions remain compacted, preserving the TCR in a closed conformation (64). This finding aligns with independent molecular dynamics simulations (65). It however contrasts with cryo-EM studies conducted in detergent environments, where this compact conformation was not observed (66–69), plausibly because detergents disrupt crucial lipid-TCR interactions. These interactions are essential for maintaining the receptor’s membrane dynamics and are insufficiently mimicked by detergents.

The importance of lipid interactions for TCR function is supported by both computational and experimental evidence. *In silico* molecular dynamics simulations suggest that TCR-lipid interactions stabilize the receptor’s resting state (70), while experimental studies using biophotonic approaches demonstrate that dephosphorylation of phosphoinositides in the plasma membrane releases the CD3ϵ cytoplasmic tail, enhancing its accessibility to LCK and facilitating signaling (71). Further evidence for the important roles of lipids for TCR functions was suggested by a study showing that cholesterol plays a key role in stabilizing the TCR’s resting state by interacting with the TCRβ transmembrane (TM) region (59, 72). This interaction is key in suppressing phosphorylation of the ζITAMs (73). In line with this, recent structural studies have identified two cholesterol molecules embedded within the TM regions of the TCR-CD3 complex (64, 67), further underscoring the importance of lipid interactions for the TCR.

Additional lipid interactions help guard the resting TCR state. For example, the BRS motifs in the CD3ϵ and ζ cytoplasmic tails bind the inner leaflet of the plasma membrane, shielding the ITAMs from premature phosphorylation and regulating their availability for downstream signaling (34, 35, 70, 71). These interactions provide a compelling regulatory mechanism, ensuring the TCR remains inactive in resting T cells. Interestingly, while some basal phosphorylation of the ζ chain can be detected in non-stimulated cells, the εITAMs appear to be fully protected from phosphorylation (35, 74). This differential “tonic” phosphorylation may be explained by the fact that the CD3ϵ cytoplasmic domain has a higher affinity for lipids than the ζ cytoplasmic domain (27). Thereby, specific tyrosines in ζ could become transiently accessible to kinases in a subset of resting TCRs (35). This basal phosphorylation of ζ is largely mediated by LCK (75).

### Ligand-engaged TCR

Cumulative evidence support that the αβ TCR-CD3 complex undergoes conformational changes upon binding to activating pMHC (64, 76–78). This structural transition results in the exposure of critical motifs within the CD3 cytoplasmic tails, allowing the simultaneous recruitment of multiple signaling proteins (2, 37, 41, 59, 62, 72, 79, 80) (**Figure 3**). It has been proposed that mechanical forces generated during the TCR-pMHC binding induce the formation of so-called catch bonds that stabilize the interaction between the TCR and agonistic pMHC, prolonging signaling (81, 82).

Although, the key role of LCK in TCR phosphorylation is broadly accepted, the mechanism of LCK recruitment to the ligand-engaged but unphosphorylated TCR remains an active area of study. As LCK is the only SFK member associated with CD4 and CD8 co-receptors, the intuitive model proposes that LCK is recruited to TCR-pMHC class I and TCR-pMHC class II via CD8 and CD4, respectively (83). However, T-cell signaling can be induced in a co-receptor-independent manner, suggesting the existence of additional mechanisms of LCK docking at the TCR-CD3 complex.

Indeed, it has been shown that the RK motif within CD3ϵ recruits LCK via its SH3 domain to the ligand-engaged TCR (2). The ϵBRS motif further enhances this process by binding LCK’s unique domain, increasing the local concentration of LCK at the TCR (1). With each TCR-CD3 complex containing two CD3ϵ chains, the recruitment of two LCK molecules may promote trans-phosphorylation, further accelerating TCR activation. However, experimental evidence supporting this hypothesis is still lacking. Not all ITAM tyrosines are equally phosphorylated, and phosphorylation of individual tyrosines has distinct functional consequences (1). For example, phosphorylation of the first tyrosine in the ϵITAM stabilizes the LCK-CD3ϵ interaction, while phosphorylation at the second tyrosine disrupts it (18). The PRS motif within CD3ϵ also contributes to LCK recruitment by binding the adaptor protein NCK, which in turn stabilizes LCK localization at the TCR (41, 42, 47, 84, 85). NCK localization at the TCR is further stabilized when the NCK SH2 domain binds to the phosphorylated second tyrosine of CD3ϵ (86), while phosphorylation of the first CD3ϵ tyrosine disrupts NCK binding (44, 86). In addition, LCK interacts with phosphotyrosine motifs in the TCR-CD3-ZAP70 complex via its SH2 domain to further amplify the signaling (87–89). The importance of the LCK SH2 domain is highlighted by the observation that LCK with mutated SH2 does not trigger TCR signaling in Jurkat cells (90).

Together, these findings suggest that multiprotein interactions, which are highly dynamic and precisely regulated, control LCK concentration nearby the TCR upon ligand binding.

Meanwhile, large membrane proteins such as the phosphatases CD45 and CD148 are excluded from the close-contact zones of TCR-pMHC contact. This steric exclusion prevents premature dephosphorylation, ensuring efficient propagation of the TCR signal (61, 91–94).

### Phosphorylated TCR and downstream signaling

Dual phosphorylation of the ITAMs by LCK creates high-affinity docking sites for the SH2 domains of ZAP70 (19, 95–97) (**Figure 3**). ZAP70’s binding follows a hierarchical pattern, with the highest affinity for the membrane-proximal ζITAM1, δITAM and γITAM (15), followed by the additional ITAMs of ζ (ζITAM2/3) and the lowest affinity for ϵITAM (97–99). Upon binding to dually phosphorylated ITAMs, ZAP70 undergoes a conformational change that repositions its SH2 domains and relieves autoinhibitory interactions within its linker region (100). This structural rearrangement primes ZAP70 for phosphorylation by LCK, fully activating ZAP70’s kinase activity (96, 97).

Once activated, ZAP70 phosphorylates the key scaffolding proteins LAT and SLP76 (101), creating platforms for recruiting additional signaling molecules necessary for downstream signal propagation (6, 102, 103). The efficiency of signal propagation depends on the stability of the TCR-pMHC interaction: a half-life long enough to sustain ITAM phosphorylation by ZAP70 is crucial for engaging downstream pathways, ultimately leading to T-cell activation, proliferation, and differentiation (104, 105).

These proximal signaling events do not occur in isolation, but rather within the specialized microenvironment of the immunological synapse. Upon TCR engagement, local calcium (Ca²^+^) concentrations surge due to the co-localization of TCRs and CRAC channels. Ca²^+^ can directly bind to lipid phosphate groups, neutralizing their charge and disrupting ionic interactions with the ϵBRS and ζBRSs. This disruption further exposes ITAM tyrosines for phosphorylation and enhances signal amplification (106). This Ca²^+^-dependent mechanism may allow bystander TCRs, not directly engaged with antigen, to become activated as well. Although monomeric pMHC alone might be sufficient to initiate proximal TCR signaling events (21, 107–109), TCR clustering within the synapse significantly enhances signal transduction by concentrating ITAMs, kinases, and other signaling molecules in close proximity (110, 111), ensuring effective downstream signal propagation.

## Role of LCK in TCR signal initiation

The conformational change model provides insights into how TCR engagement with its cognate pMHC leads to the exposure of the ITAMs as substrates for tyrosine phosphorylation. SFKs, a group of non-receptor tyrosine kinases, play a pivotal role in this process. These kinases can be divided into two subgroups: SRC-A and SRC-B. Among them, LCK, a member of the SRC-B group, is the predominant SFK in T cells. FYN, an SRC-A member, can partially rescue LCK deficiency in T cells. Both LCK and FYN are myristoylated and palmitoylated, which mediates their association with the inner membrane leaflet (6). While FYN contributes to TCR signaling, its role is less prominent than LCK for ITAM phosphorylation (112, 113). The role of FYN is mostly highlighted in LCK-deficient mice, where it facilities residual TCR signaling during thymic development and partially rescues the formation of mature T cells (49, 50). Interestingly, FYN has been proposed as a potential negative regulator of TCR signaling, acting through phosphorylation of CSK-binding protein (CBP, also known as PAG) (114). Additionally, FYN has been reported to trigger cytoskeletal rearrangements following TCR triggering (115). Further experimental effort is needed to comprehend the overlapping and unique roles of LCK and FYN in T cells. In this review, we will further focus on LCK, which is the major SFK in TCR signal initiation.

### The regulation of LCK

LCK activity is highly regulated by maintaining a balance between its active and auto-inhibited states. Two key tyrosine residues control this balance: the activating tyrosine in the catalytic domain (Y394 in humans), which must be phosphorylated for full kinase activity, and the inhibitory tyrosine in the C-terminal domain (Y505 in humans). Phosphorylation of Y505 facilitates an intramolecular bond with the SH2 domain, locking LCK in a closed, inactive conformation (116, 117).

The equilibrium between these active and inactive states is highly dynamic and regulated by a network of phosphatases and kinases, including the tyrosine kinase CSK, which phosphorylates Y505 (118), the membrane-bound protein tyrosine phosphatases CD45 and CD148 (75, 119, 120), as well as cytosolic phosphatases such as PTPN6 (SHP1) (121), PTPN22 (122), and the PEST domain-enriched tyrosine phosphatase (PEP) (123, 124). Furthermore, it has been recently proposed that LCK binds to the homodimeric receptor CD146, a cell-adhesion molecule, which leads to LCK’s trans-autophosphorylation and activation (125). Together, these regulatory mechanisms ensure that LCK activity, and consequently TCR phosphorylation, remains tightly controlled in the absence of ligand engagement, preventing unwanted activation.

However, it is unclear how TCR engagement would mechanistically instruct LCK’s catalytic activity beyond the recruitment of active LCK molecules close to the TCR. A substantial proportion of LCK is constitutively active in resting T cells (126–128). Some of this active LCK is located near the TCR, even in the receptor’s resting state, poised for immediate engagement when the TCR transitions to its prime state (**Figure 3**) (2). Whether TCR triggering increases the pool of active LCK and what the mechanism and kinetics would be is still a subject of debate (126, 129–131). A straightforward explanation, pending experimental confirmation, is that this pre-activated LCK directly phosphorylates the ligand-bound TCR without requiring further activation of additional LCK molecules. Such a mechanism would enable the rapid initiation of TCR signaling.

### Co-receptor bound LCK is not essential for TCR signaling

LCK exists either as a free pool or in association with the co-receptors CD4 or CD8 (5, 132). As LCK is the dominant SFK in T-cell activation, and the only family member capable of interacting with the co-receptor CD4 and CD8, an intuitive model of TCR signaling postulated that CD4 or CD8 deliver LCK to the TCR-pMHC complex, serving as the primary trigger of TCR signaling (133).

**Figure 4:**
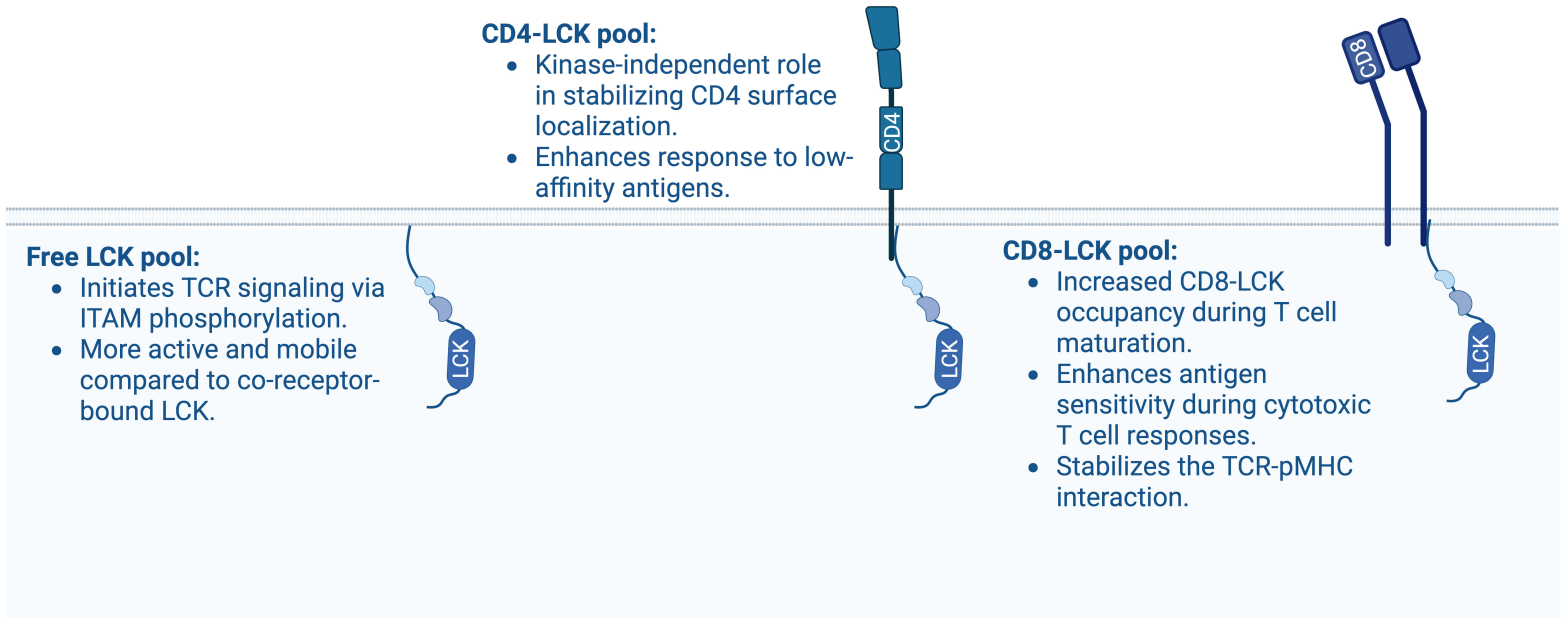
Different pools of LCK and their potential characteristics. Depicted is free LCK, CD4-associated LCK and CD8-associated LCK and their main contributions to αβ TCR-CD3 signal transduction.

However, this paradigm has evolved with evidence showing TCR signaling can occur independently of co-receptor engagement. Experiments using anti-CD3 antibodies, CARs, or MHC class I mutants with a 10-fold weaker CD8-affinity than WT (134), demonstrate that TCR signaling does not strictly require co-receptor interaction. Additionally, unconventional T cells, which recognize non-MHC antigens, naturally bypass co-receptor involvement. Collectively, these findings reveal that co-receptor aggregation of the TCR is not an essential mechanism for TCR signal initiation (**Figure 4**). While co-receptor-bound LCK is not strictly required for TCR triggering, it plays a significant role in enhancing the sensitivity of TCR signaling, especially in response to antigens with suboptimal affinity (135–138). Recent insights into the physiological roles of CD4-LCK and CD8-LCK were addressed in a landmark study using knock-in mice expressing a LCK CA mutant (C20A and C23A), which does not interact with CD4 or CD8 (137). Although these mice showed reduced positive selection, their phenotype was relatively mild especially in terms of cytotoxic responses to viral infections and tumors.

In sharp contrast, LCK-deficient or kinase-dead LCK models exhibit severe impairments (49, 137, 139–141). This suggests that free LCK can largely compensate for the loss of co-receptor-associated LCK *in vivo* (137).

### CD4-LCK: Co-receptor stabilization and fine-tuning of TCR signaling

LCK associates with CD4 through a zinc clasp formed by cysteine-rich motifs in the CD4 cytoplasmic tail and the N-terminal part of LCK (5, 132, 142). A large proportion of CD4 molecules are coupled with LCK in developing DP thymocytes (136), with a modest increase in CD4-LCK coupling observed during CD4^+^ T-cell maturation (143). This is likely due to the higher affinity of LCK for CD4 compared to CD8 (132) and the expression of a truncated CD8 isoform unable to bind LCK (144), leading to preferential sequestration of LCK to CD4 in the DP stage (145). Estimates from co-immunoprecipitation studies suggest that between 36% and 80% of CD4 is bound to LCK in primary murine CD4^+^ T cells from lymph nodes (143, 146). Given the relatively weak co-receptor-LCK interaction and possible artifacts upon cells lysis with detergents, methods that do not require cell lysis, such as *in situ* proximity ligation assays (PLA) or quantitative single-molecule microscopy may provide more accurate occupancy measurements.

Genetic disruption of the CD4-LCK interaction reduces CD4^+^ SP thymocyte numbers and impairs helper T-cell functions. However, some CD4^+^ T-cell functions can be rescued by kinase-dead CD4-bound LCK, suggesting a kinase-independent role for CD4-LCK in stabilizing CD4 localization at the plasma membrane (137). Similarly, chimeras formed by CD4 fused to kinase-deleted LCK were shown to be more efficient at supporting full activation of a CD4-dependent T-cell hybridoma than the full-length LCK chimeric fusion protein (147). This kinase-independent stabilization of CD4 by LCK is essential for regulating the surface levels and trafficking of CD4 in primary T cells, as previously demonstrated in transgenic non-lymphoid cell lines (148, 149). This suggests that CD4 has LCK-independent or at least kinase-independent roles in T-cell development and signaling.

While CD4-bound LCK is not strictly required for TCR signaling, it enhances signal transduction (136, 150), fine-tunes antigen sensitivity (137), and stabilizes CD4 at the cell surface (137, 148, 149). This underscores the modulatory role of co-receptor-associated LCK in antigen sensitivity and signal amplification.

### CD8-LCK: TCR-pMHC stabilization and modulation of cytotoxic T-cell response

LCK associates with CD8 in a manner similar to CD4, though CD8 and CD4 differ structurally and functionally. CD8 exists in two isoforms, CD8α and CD8β, which form homo- or heterodimers. Conventional CD8^+^ αβ T cells express the CD8αβ heterodimer, while γδ T cells and NK cells primarily express the CD8αα homodimer (151). LCK associates with the CD8α subunit (152). Despite the potential for CD8αα to bind two LCK molecules, CD8β enhances the interaction between LCK and CD8α, raising questions about how much LCK associates with CD8αα homodimers (153).

In DP thymocytes, CD8-bound LCK is less abundant compared to CD4-bound LCK (136). However, during T-cell maturation, the proportion of CD8-bound LCK increases, correlating with enhanced homeostatic responses to self-antigens in mature CD8^+^ T cells (136, 143).

Although CD8-LCK is essential for fine-tuning cytotoxic T-cell responses to weak antigens in a kinase-dependent manner, CD8-bound LCK appears largely dispensable for the *in vivo* development of cytotoxic T cells and anti-viral/anti-tumor responses (137). However, supraphysiological CD8-LCK stoichiometry, in T cells expressing a chimeric CD8.4 co-receptor, consisting of the extracellular CD8α domain and the intracellular LCK-binding CD4 part, has relatively strong effects on enhancing the positive selection of very weakly self-reactive thymocytes (154), decreasing antigen affinity threshold for negative selection (136), and inducing antigen-independent memory-like T cells (also known as virtual memory T cells) (155). This suggests that the relatively minor role of the CD8-LCK interaction *in vivo* might be, at least partially, caused by the relatively low CD8-LCK stoichiometry, which makes free LCK dominant even in WT CD8^+^ T cells.

### Implications for thymic selection and MHC restriction

LCK delivered to the TCR by CD4 or CD8 during thymic selection was proposed to impose MHC restriction on the developing αβ TCR repertoire (156). Supporting this theory, TCRs in mice lacking both, co-receptors and MHC (quad-deficient mice), develop non-pMHC-specificities resembling those of antibodies (145, 157) and are enriched for CD1d-restricted iNKT receptors (137). However, the co-receptor-LCK interaction is not strictly required for MHC restriction, as TCR transgenic monoclonal MHC class I- or II-restricted thymocytes develop normally in the LCK CA mice (137). Given the prominent role of CD8 and CD4 co-receptors in the lineage commitment, these observations reveal LCK-independent roles of the co-receptors in T-cell triggering, probably by stabilizing the pMHC-engaged TCR (158, 159). Although a role of CD4 in TCR-pMHCII stabilization has been strongly questioned based on its low affinity to isolated pMHCII complexes (150), recent evidence shows that the CD4-pMHCII affinity increases substantially when the pMHC is engaged by a cognate TCR (160).

### Interplay between free LCK and co-receptor-bound LCK in TCR signal initiation

The role of free LCK *versus* co-receptor-bound LCK in TCR signaling has been a subject of significant interest. Current evidence suggests that free LCK is more efficient than CD8-bound LCK in initiating ITAM phosphorylation (161) and that CD8-bound LCK is recruited only later, stabilizing the whole TCR-pMHCI-CD8-LCK complex (158, 161). This model is supported by studies showing that free LCK moves faster and is more associated with the TCR at the steady-state in thymocytes than co-receptor-bound LCK (128). These findings indicate that free LCK may be particularly critical for triggering the ligand-engaged TCR in contexts where co-receptors do not interact with the ligand, such as during antibody stimulation, in CAR T cells, or in unconventional T cells that recognize non-MHC antigens. Mathematical modeling further emphasizes the role of co-receptor-bound LCK in signal amplification rather than signal initiation. Recruitment of CD4-LCK enhances TCR phosphorylation threefold compared to free LCK alone. However, when recruited to a pre-phosphorylated TCR, this effect is amplified 30- to 40-fold, additionally suggesting that co-receptors may act to boost signaling after the initial trigger has been established (162).

As mentioned above, co-receptor-bound LCK is crucial for signaling triggered by low-affinity ligands. These ligands bind shortly to the TCR and may require additional mechanisms to recruit sufficient LCK to complete all phosphorylation steps before the ligand dissociates. The kinetic proofreading model highlights the need for all proximal signaling steps to occur within the limited duration of the TCR-pMHC interaction to enable downstream signaling (104, 105, 135, 163–165). The cooperative engagement of free and co-receptor-bound LCK may reduce the time required for LCK to access and phosphorylate the ITAMs. This is supported by studies in compound heterozygous mice expressing the LCK CA mutant, which is unable to bind CD8 (free LCK only) and a kinase-dead LCK mutant bound to CD8. These mice exhibit weaker responses to suboptimal antigens than homozygous LCK WT or even LCK CA mice (137), suggesting that the kinase activity of CD8-LCK facilitates TCR signaling when the duration of the TCR-pMHC bond is the limiting factor.

During T-cell development, CD4 and CD8 co-receptors are highly expressed in DP thymocytes, ensuring preferential selection of TCRs recognizing MHC antigens and enabling discrimination between self and non-self MHC molecules. These high co-receptor levels reduce the availability of free LCK for TCRs that recognize non-MHC ligands, preventing the positive selection of DP thymocytes bearing such TCRs (166) and lowering the relative abundance of positively selected iNKT cells (137). In contrast to DP cells, SP thymocytes and mature T cells express only one co-receptor that matches the MHC-restriction of their TCR. In these cells, it is still not clear whether and how the roles of free LCK and co-receptor-bound LCK differ. Studies show that LCK-deficient primary T cells expressing only the LCK CA mutant, which cannot bind co-receptors, exhibit weaker ζ-chain phosphorylation than cells expressing WT LCK, suggesting that both free and co-receptor-bound LCK pools are critical for robust TCR-CD3 signal transduction (161).

Despite substantial advances, key aspects of free *versus* co-receptor-bound LCK remain unresolved. Open questions include the precise stoichiometry of CD8- and CD4-bound LCK specially in mature conventional T cells (136, 143, 167), potential differences in the kinase activities of free *versus* co-receptor-bound LCK (128, 168), and whether these pools perform distinct mechanistic roles during TCR signal initiation (137, 161). The balance between free and co-receptor-bound LCK reflects a finely tuned mechanism that ensures both sensitivity and efficiency in TCR signaling. Relative contributions of these LCK pools may depend on factors such as LCK and co-receptor expression levels, the proportion of LCK coupled to the co-receptors, ligand affinity, and the presence of phosphatases. Understanding this balance is critical for comprehending TCR signaling dynamics and could provide insights into therapeutic strategies for modulate immune responses in cancer immunotherapy and other immunomodulatory contexts.

## Mechanism of γδ TCR signaling initiation

γδ T cells represent a unique subset of T lymphocytes, characterized by their expression of the γδ TCR, which is formed from diverse TCRγ (e.g., human Vγ2-5/7/8/11; mouse Vγ1-7) and TCRδ chains (e.g., human Vδ1-3/5; mouse Vδ1/2/4-6) (169). Although they make up only 0.5–5% of the peripheral blood T-cell population, γδ T cells are more abundant in peripheral tissues, where they play essential roles in immune surveillance and early responses to infections and tumors (170, 171).

In contrast to αβ T cells, which are restricted to recognizing pMHC complexes, γδ TCRs are capable of recognizing a broad range of antigens independently of MHC presentation (172–174). These antigens include phospho-antigens, lipids, and stress-induced molecules expressed by infected or cancerous cells (7, 175). This ability to engage non-peptide antigens without MHC involvement suggests that γδ TCRs initiate signaling through mechanisms that are distinct from those of αβ T cells, likely involving specialized kinases and signaling pathways. The following sections will examine these unique signaling mechanisms of γδ T cells, highlighting how they differ from the signal initiation seen in αβ T cells.

### Structure of the γδ TCR-CD3 complex

Similar to αβ TCRs, γδ TCRs assemble with the CD3 signaling complex. In humans, this includes CD3ϵγ, CD3ϵδ, and ζζ dimers, while in mice, the complex features two CD3ϵγ dimers and ζζ (176). Structural and functional differences between human and mouse γδ T cells complicate direct translation of findings between species.

**Figure 5:**
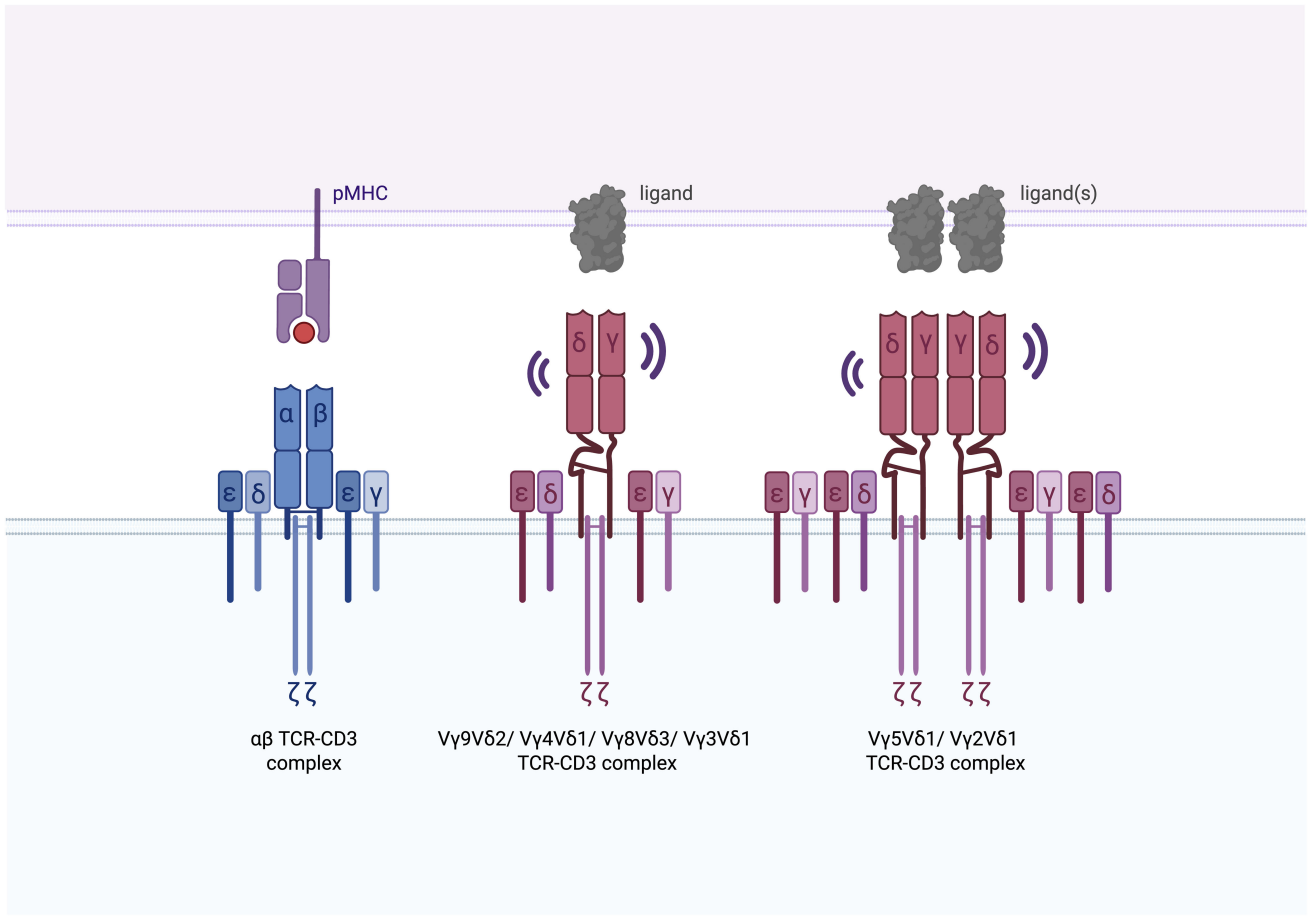
Assembly of human αβ and γδ TCR-CD3 complexes. The αβ TCR-CD3 complex is tightly assembled, with the ectodomains of the TCRα and TCRβ chains closely packed against the ectodomains of CD3ε, CD3γ, and CD3δ. The Vγ9Vδ2 TCR, although maintaining the same stoichiometry as the αβ TCR-CD3 complex, exhibits notable conformational flexibility in its TCRγδ ectodomains and connecting peptides, akin to the dynamic behavior observed in Fabs and hinge regions of membrane-bound immunoglobulins. Evidence supports that Vγ4Vδ1, Vγ8Vδ3, and Vγ3Vδ1 TCRs also assemble as monomers (179, 180). In contrast, the Vγ5Vδ1 and Vγ2Vδ1 TCR assemble as dimers (179).

Key structural differences between αβ and γδ TCRs lie in their constant regions and connecting peptides, which influence receptor assembly, surface orientation, and charge distribution (177, 178). For example, the extracellular domains of γδ TCRs exhibit greater conformational flexibility compared to αβ TCRs (179, 180). This increased flexibility may enable γδ T cells to recognize a wider range of ligands with varying sizes and structures. A recent cryo-EM study uncovered variability in γδ TCR assembly based on the Vγ chain usage (179) (**Figure 5**). For instance, the Vγ9Vδ2 TCR appears monomeric and exhibits substantial conformational flexibility in its γδ TCR ectodomains and connecting peptides, resembling the dynamics of Fabs (fragment antigen-binding) regions and hinge linkers of membrane-bound Igs. FLIM-FRET analysis further supports this, showing that other γδ TCRs, such as Vγ4Vδ1, Vγ8Vδ3, and Vγ3Vδ1, also exist as monomers (179). In contrast, the Vγ5Vδ1 TCR forms a dimeric structure, which is crucial for its activation, and FLIM-FRET analysis indicates that Vγ2Vδ1 TCRs also assemble as dimers (179) (**Table 2**). These structural differences underscore the unique assembly mechanisms and flexibility of γδ TCRs compared to the more rigid αβ TCR complexes (**Figure 5**). While cryo-EM studies have provided valuable insights into γδ TCR assembly (179, 180), future studies employing native-like nanodiscs instead of detergent environments could further illuminate the diverse conformations of γδ TCRs. Although these structural variations suggest that the mechanisms of TCR activation may not be fully conserved between αβ and γδ TCRs, some parallels might exist. For instance, cholesterol has been shown to restrain signaling in both Vγ9Vδ2 and Vγ5Vδ1 TCRs, possibly by altering the conformation of the ζ chains (179). This suggests that certain regulatory processes may be shared between αβ and γδ TCRs, by conservation of key residues across TCR subtypes and species. The following sections will further explore both shared and unique mechanisms of activation between αβ TCRs and γδ TCRs.

**Table 2:** Summary of current knowledge on γδ T-cell subsets.

Subset	Species	Vδ usage mouse	Vδ usage human	Suggested assembly	Genetic evidences of structural changes	Biochemical evidences of structural changes	Proximal kinases
Vγ1	mouse	Vδ2/4/5/6	-	-	no	-	SYK
Vγ1^+^ γδ NK T		Vδ1	–	–	yes	–	–
Vγ2	human/mouse	Vδ5	Vδ1/3	dimeric	-	-	-
Vγ3	human/mouse	Vδ1	Vδ1/3	monomeric	–	–	LCK
Vγ4	human/mouse	Vδ1/2/4/5	Vδ1/5	monomeric	yes	no	No LCK SYK No ZAP70
Vγ5 (DETC)	human/mouse	Vδ1	Vδ1	dimeric	no	–	ZAP70 or SYK
Vγ5^+^ (non-DETC)	mouse	Vδ1	Vδ1	-	yes	-	-
Vγ6	mouse	Vδ1	–	–	–	–	BLK No LCK ZAP70 & SYK
Vγ7	mouse	Vδ2/4/5/6	-	-	no	-	-
Vγ8	human	–	Vδ1/3	monomeric	–	–	–
Vγ9	human	-	Vδ2	monomeric	-	no	LCK
Vγ11	human	–	–	–	–		–

This table outlines the characteristics of γδ T-cell subsets in mice (purple) and humans (blue), including their Vγ-Vδ chain pairings, assembly as monomers or dimers, and genetic and biochemical evidence for structural changes. It also highlights the kinase dependencies involved in proximal γδ TCR signaling, including subsets that rely on LCK, ZAP70 or SYK (or both), and those that function independently of LCK. “-” indicates that the information is unknown to date.

### Conformational states of the γδ TCR

In αβ T cells, the binding of pMHC induces the stabilization of TCR conformational states exposing the signaling motifs within the CD3 complex. Mice with mutations in the CD3ϵ stalk region (ϵC80G), which prevents the exposure of the PRS motif upon αβ TCR engagement (181), demonstrate that this transition is essential for αβ T-cell development. Whether this mutation solely prevents PRS exposure or also impairs the switch of the αβ TCR to its active state is to date unknown. Interestingly, this mutation differentially affects γδ T-cell subsets, suggesting that γδ TCRs may not represent a single, uniform receptor, but rather a collection of receptors, each with distinct activation mechanisms (**Table 2**). For instance, Vγ1^+^, Vγ7^+^ and Vγ5^+^ (non-dentritic epidermal T cells (DETC)) γδ T cells develop normally in ϵC80G mice, while Vγ4^+^ and Vγ5^+^ (DETC) subsets are reduced (181). Additional evidence for this diversity among γδ TCRs comes from a study showing that the human Vγ9Vδ2 TCR, expressed in a Jurkat cell line, does not expose the PRS upon stimulation with its endogenous ligand (182), which was also true for the T22-specific murine G8 (Vγ4^+^) and KN6 γδ TCR (182, 183). Together, these findings highlighting the heterogeneous nature of γδ TCRs and their distinct activation mechanisms.

### Proximal γδ TCR-CD3 signal transduction

γδ TCR signaling is also initiated by the phosphorylation of CD3 ITAMs, likely mediated by SFKs. Inhibition of CSK, a negative regulator of SFKs, enhances ERK phosphorylation in γδ T cells, underscoring the importance of SFKs in γδ TCR signaling (184). Unlike αβ TCR signaling, which predominantly relies on LCK, evidence suggests that different γδ T cell subsets may rely on distinct SFKs.

γδ T cells express significantly lower levels of LCK compared to αβ T cells (185). Moreover, γδ T cells are present in LCK-deficient mice, which lack αβ T cells, indicating that LCK is not essential for all γδ T-cell subsets (186). Supporting this, patients with loss-of-function mutations in LCK exhibit a drastic reduction in αβ T cells, yet a relative increase in γδ T cells, suggesting that certain γδ T-cell populations are LCK-independent (187, 188). Notably, in one patient, Vδ1^+^ γδ T cells accumulated, whereas Vδ2^+^ γδ T cells were reduced, indicating that Vδ2^+^, but not Vδ1^+^, γδ T cells may depend more on LCK (188).

In mice, IL-17-producing Vγ6^+^ and Vγ4^+^ γδ T cells lack LCK expression and can function without it, consistent with earlier findings that LCK is dispensable for the development of these subsets (189–191). In contrast, Vγ3^+^ T-cell development is severely impaired in *Lck*^-/-^, *Lck*/*Fyn*^-/-^ and *Cd45*^-/-^ mice (49, 192) and murine CD27^+^ γδ thymocytes, which are primed to differentiate into IFNγ-producing γδ T cells, exhibit high LCK expression (190). Global gene expression analyses reveal that the B cell-specific lymphoid kinase (BLK) is expressed in murine γδ T cells but not in αβ T cells, mediating the development of Vγ6^+^ IL-17-producing γδ T cells (193, 194), indicating that other SFKs beyond LCK may play a role in γδ TCR signaling.

γδ TCR signaling strength has been linked to distinct effector fates in mice. Strong γδ TCR signaling promotes the differentiation of IFNγ-producing subsets, predominantly Vγ1^+^, Vγ5^+^, and Vγ7^+^ γδ T cells (195, 196), which likely depend on LCK. In contrast, weaker γδ TCR signaling favors the development of IL-17-producing subsets (majority of Vγ4^+^ and Vγ6^+^ γδ T cells) (195, 196), which may rely on alternative SFKs like BLK.

How SFKs are recruited to the γδ TCR presents an intriguing question, especially since most γδ T cells do not express the co-receptors CD4 or CD8 and recognize their ligands without the participation of CD4 or CD8. This suggests that alternative mechanisms, potentially involving CD3 cytoplasmic motifs such as the BRS or RK motif, facilitate the SFK recruitment. If these motifs are functional in γδ T-cell subsets needs to be addressed in the future. While ZAP70 is essential for αβ T-cell development, some γδ T cells persist in ZAP70-deficient mice, suggesting that ZAP70 is not universally required for all γδ subsets (197). Consistent with this, studies using *Zap70*^-/-^, *Sykb*^-/-^, and *Zap70*/*Sykb*^-/-^ double KO mice show that SYK is required for the generation of neonatal Vγ1^+^ and Vγ4^+^ γδ T cells, while Vγ5^+^ cells rely on either ZAP70 or SYK, and Vγ6^+^ cells depend on both (198). Given that SYK activation is less dependent than ZAP70 on SFKs (199, 200), it is likely that LCK-dependent γδ T-cell subsets preferentially use ZAP70, whereas SYK-dependent subsets do not rely on LCK. However, the γδ T cells existing in the different models do not fully support this (oversimplifying) idea (**Table 2**) and further investigation is needed.

It has been proposed that γδ TCR signal initiation by nonclassical MHC class 1b antigens requires the exclusion of phosphatases (CD45) from the vicinity of the TCR, as consequence of cell-cell close contact zones (183). This adds to the idea that tipping the equilibrium between kinases and phosphatases in the vicinity of the engaged-TCR aids to activation. Whether this is the case for all γδ TCRs and all γδ T-cell subsets needs to be elucidated.

Understanding the interplay between the different SFKs and γδ TCR downstream signaling pathways will be essential for fully elucidating the molecular mechanisms governing γδ T-cell function and their potential therapeutic applications.

### γδ T cells in cancer

The role of γδ T cells in tumor surveillance is well-established. Studies in mice have shown that the absence of γδ TCRs increases susceptibility to certain cancers (201). In humans, the presence of γδ T cells is the most favorable survival predictor across 25 malignancies and most solid tumors (202). Their presence at tumor sites correlates with improved clinical outcomes in colorectal, gastric, and head and neck cancers (203–205). However, the role of human γδ T cells in cancer appears to be influenced by their subset-specific characteristics. Vδ1^+^ T cells, predominantly found in tissues, form diverse subsets by pairing with various Vγ chains. While some Vδ1^+^ T cells recognize glycolipids presented by CD1c and CD1d molecules (206, 207), the specific TCR antigens for most Vδ1^+^ T cells remain unknown. These cells exhibit potent anti-tumor activity in colorectal cancer, multiple myeloma, and chronic lymphocytic leukemia (208–210). However, they can also exert immunosuppressive effects (211–213). In contrast, the human Vδ2^+^ subset exclusively pairs with the Vγ9 chain to form Vγ9Vδ2 T cells, the predominant γδ T-cell population in peripheral blood. These cells recognize phosphoantigens derived from pathogens or tumor cells (214) and have been extensively studied in clinical settings due to their strong anti-tumor activity (215–220). The roles of human Vδ3^+^ and Vδ5^+^ γδ T cells in cancer remain less characterized (171, 221–224).

Emerging therapeutic strategies aim to leverage the unique properties of γδ T cells. These include the development of bispecific cell engagers, adoptive transfer of expanded Vδ1^+^ or Vδ2^+^ T-cell populations, and genetic engineering approaches such as CAR γδ T cells or αβ T cells engineered to express specific γδ TCRs (225). Recent clinical trials exploring γδ T cells in cancer immunotherapy have shown promising, but still modest, results emphasizing their potential as a tool in cancer treatment (171, 213, 226).

To fully harness the therapeutic potential of γδ T cells, a deeper understanding of the molecular mechanisms underlying γδ TCR signaling is essential. In particular, the proximal signaling steps leading to γδ T-cell activation require further investigation. The following sections will examine how insights into proximal αβ TCR signaling events have driven the development of novel therapeutic approaches (4), providing a framework to inspire innovations in γδ T cell-based therapies.

## Harnessing the TCR signaling potential for cancer immunotherapy

**Figure 6:**
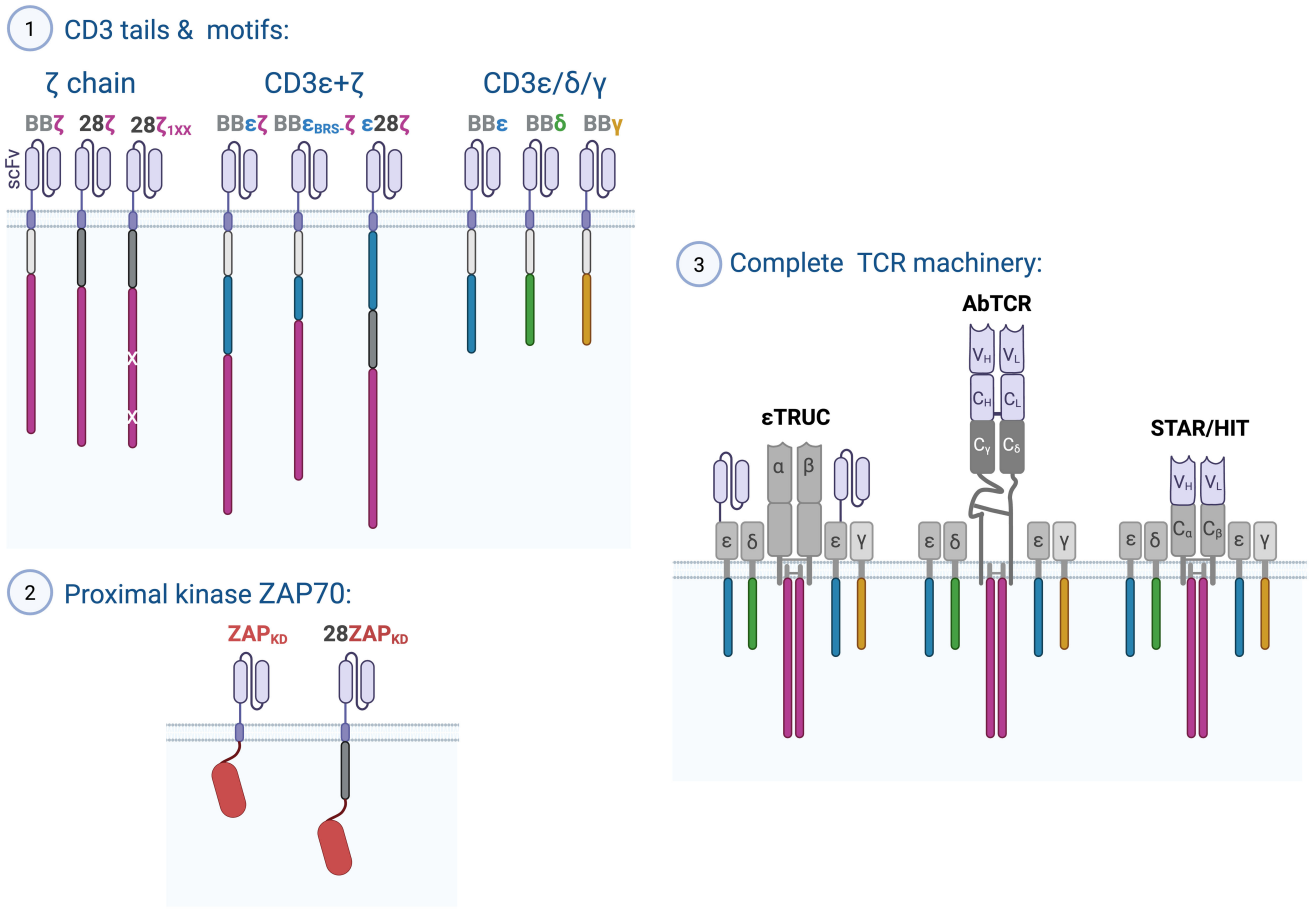
Harnessing proximal TCR-CD3 signaling mechanisms to improve cancer immunotherapy. Depicted are chimeric receptor constructs which in pre-clinical models have shown potent anti-tumor efficacy. Three strategies have been followed to improve FDA-approved second generation CARs containing a co-stimulatory domain derived from 4-1BB or CD28 and a signaling domain derived from the ζ chain: (1) Reducing ζITAMs (1XX), incorporating CD3ϵ in addition to ζ, or exchanging the ζ chain for CD3ϵ, CD3δ or CD3γ. (2) Using the kinase domain (KD) of ZAP70 alone or in combination with a co-stimulatory domain from CD28, and (3) generating TCR-like chimeric receptors to harness the complete CD3 complex with all its motif for signal transduction. The εTRuC where a single chain variable Fragment (scFv) is fused to the CD3ε ectodomain, the AbTCR, where the Fab fragment is fused to the ectodomains of the γδ heterodimer, as well as STAR/HIT designs where antibody-derived variable regions replace the variable regions of TCRαβ domains.

Discoveries in TCR signaling have the potential to profoundly influence the design of CARs and TCR-like chimeric receptors, advancing their effectiveness in cancer immunotherapy. To date, the approaches to improve T-cell activation and anti-tumor activity can be broadly categorized into three main strategies: (1) incorporating specific signaling motifs from CD3 chains into CAR designs, (2) using proximal kinases at part of a CAR construct, and (3) leveraging the complete TCR complex to optimize its signaling potential, including structural regulation and the diverse functions of the CD3 cytoplasmic domains (4) (**Figure 6**).

### ζ-based second generation CARs

CARs are engineered receptors that redirect T cells to recognize and kill tumor cells. A CAR typically consists of three main components: an extracellular binding domain, generally a single-chain variable fragment (scFv) derived from an antibody, that recognizes the target antigen; a TM region anchoring the receptor in the membrane; and intracellular signaling domains responsible for activating the T cell. In the following section, we focus on different intracellular signaling domains, without delving into other structural advances such as in the binding domain or TM region of CAR designs.

Early CAR designs were based on the ζ chain of the TCR, known for its strong signaling capacity. These first-generation CARs successfully drove T-cell proliferation, cytokine production, and cytotoxicity *in vitro* and *in vivo* (227, 228). However, their limited persistence *in vivo* hindered their therapeutic potential. To address this, second-generation CARs incorporated co-stimulatory domains originating from co-stimulatory receptors such as 4-1BB (also known as CD137 and TNFSFR9) or CD28, alongside the ζ chain (henceforth referred to as BBζ and 28ζ CARs, respectively; **Figure 6**). This innovation significantly improved CAR T-cell persistence and tumor-eradicating capability (229). Notably, CD28 contains two basic-rich stretches (BRSs) that interact with the plasma membrane and LCK, whereas 4-1BB has only one juxtamembrane BRS. It was shown that the higher signaling intensity of 28ζ CARs, compared to BBζ CARs, results from constitutive LCK association with CD28 (230). However, recent findings also revealed that 28ζ CAR signaling can occur independent of LCK, relying instead on the kinase FYN, whereas both TCR and BBζ CAR signaling are LCK-dependent (231).

Most CAR designs retain the ζ chain, assuming its multiple ITAMs would amplify signaling. However, it has become evident that reducing ITAM numbers may actually improve therapeutic outcomes. CARs with a single functional ζITAM (1XX CARs) demonstrated superior anti-tumor efficacy compared to those with two or three ITAMs in a CD19^+^ tumor *in vivo* model (232). These 1XX ITAM CARs promote a favorable memory T-cell phenotype with enhanced persistence (232), supporting the notion that reducing signaling potency can reduce activation-induced cell death (AICD) and exhaustion, particularly in cases of high tumor antigen expression. These promising findings have already inspired clinical trials using the 1XX CAR format (233, 234).

### CARs employing alternative CD3 chains

Recent advances in CAR design have focused on incorporating signaling domains beyond the ζ chain ITAMs, particularly leveraging the unique functionalities of CD3 subunits such as CD3ϵ, CD3δ, and CD3γ (**Figure 6**). These efforts aim to enhance CAR T-cell efficacy, persistence, and safety.

For instance, BBεζ CARs, which combine a 4-1BB co-stimulatory domain with CD3ϵ and ζ chains, exhibit superior anti-tumor activity in leukemia models compared to BBζ CARs (2, 8) by recruiting LCK through the RK motif (2). Similarly, the BRS motif of CD3ε modulates interactions with LCK and p85, reducing cytokine production while increasing T-cell persistence for a ε28ζ CAR (CD3ε, CD28, and ζ chain) relative to traditional 28ζ CARs (1). CARs incorporating the CD3ϵ cytoplasmic tail together with a CD28 co-stimulatory domain (28ϵCAR) showed increased efficacy in a solid tumor models while minimizing inflammatory cytokine release compared to a 28ζ CAR (235). Additionally, CD3ϵ-containing CARs regulate LCK activity through the recruitment of the kinase CSK via the mono-phosphorylated ITAM of CD3ϵ, providing negative feedback control (1). Mutations to the BRS, RK, or ITAM motifs in CD3ε-based CARs impaired CAR T-cell function *in vitro*, underscoring their critical role in optimizing CAR signaling (3).

Expanding on this, CARs incorporating signaling domains from CD3δ or CD3γ in the context of the 41BB co-stimulatory domain show enhanced anti-tumor efficacy, reduced tonic signaling, and lower cytokine secretion compared to BBζ CARs (3). CD3δ, for instance, recruits SHP1 through monophosphorylation of the second ITAM tyrosine, providing regulatory control and promoting stem-like T-cell properties and CAR functionality (3). Furthermore, mutations in the di-leucine motifs of δCARs and γCARs boost surface expression, T-cell cytotoxicity and activation, highlighting the role of this motif in regulating CAR functionality (3).

These findings underscore the critical importance of understanding proximal TCR signaling to guide the rational design of CARs. While current FDA-approved CAR therapies rely on second-generation ζ chain constructs, emerging evidence suggests that alternative designs incorporating CD3ϵ, δ, or γ signaling domains may provide significant advantages, including enhanced efficacy, reduced tonic signaling, and improved T-cell persistence (**Figure 6**). The next frontier in CAR T-cell optimization could lie in harnessing the unique properties of these underexplored CD3 subunits. Clinical trials will be essential to determine whether these novel CAR constructs can surpass the performance of current therapies. Moreover, the strategic combination of different CD3 signaling domains (1, 2) represents a promising avenue to further refine CAR designs, potentially unlocking more durable and effective cancer immunotherapies.

### CARs coupled to downstream signaling proteins

Alternative CAR formats have been developed to bypass the earliest TCR signaling steps, ITAM phosphorylation, by directly integrating downstream signaling proteins (236, 237). These herein termed bypassCARs are coupled directly to molecules such as LCK, FYN, the kinase domain (KD) of ZAP70 (ZAP70_KD_), LAT, SLP76, or PLCγ1, without including any co-stimulatory domains (238). Among these designs, the ZAP70_KD_-bypassCAR (**Figure 6**) demonstrated superior tumor control in a solid-tumor mouse model compared to a conventional BBζ CAR (238). An additional study demonstrated that adding the CD28 signaling domain before the ZAP70_KD_ (**Figure 6**) resulted in a construct capable of inducing tumor remission comparable to a BBζ CAR and more durable than a 28ζ CAR in a Nalm6 tumor mouse model (239).

These findings highlight the potential of bypassCARs to overcome limitations in traditional designs, but further studies are needed to optimize their efficacy, evaluate possible undesired tonic signaling and thereby, premature exhaustion, and evaluate their clinical relevance across diverse tumor models.

### TCR-like chimeric receptors

TCR-like chimeric receptors represent an exciting frontier in engineered T-cell therapies, leveraging the entire TCR-CD3 complex for signal transduction instead of isolated CD3 chains. Ideally, these receptors function independently of MHC.

One such approach, TCR-fusion constructs (TRuCs), involves fusing a scFv to the extracellular domain of CD3ϵ. TRuCs have shown superior efficacy in both hematopoietic and solid tumor models compared to second-generation BBζ and 28ζ CARs (240, 241) and are currently being investigated in clinical trials with promising interim results (242).

In another strategy, antibody-TCRs (AbTCRs), a full, anti-tumor Fab-fragment is fused to the constant domains of the TCRγ and TCRδ chains, creating new chimeric receptors that outperformed BBζ and 28ζ CARs in controlling B-cell tumors *in vivo*. These AbTCR-T cells also exhibited reduced cytokine production and exhaustion marker levels compared to 28ζ CAR T cells (243). Using the constant domains of TCRγ and TCRδ prevents undesired pairing with endogenous TCRα and TCRβ chains, reducing the risk of generating off-target specificities that could lead to autoimmunity. However, how these AbTCRs are coupled to proximal signaling pathways has yet to be elucidated, therefore, the exact mechanisms underlying their superior performance remain unclear.

Synthetic TCR and Antigen Receptors (STARs) (244) and HLA-independent TCRs (HITs) (245) replace the variable regions of TCRα and TCRβ with antibody-derived variable regions. In preclinical mouse models, STAR and HIT T cells provided superior tumor control compared to 28ζ CARs, especially in tumors with low antigen expression (245). STAR and HIT T cells were also more effective at infiltrating solid tumors, less prone to exhaustion, and persisted longer than BBζ and 28ζ CARs. Interestingly, STAR and HIT T cells produced similar or even higher amounts of cytokines than BBζ and 28ζ CARs upon tumor recognition, likely due to their enhanced sensitivity (243, 245, 246). This can eventually be a drawback for these receptors, as increased cytokine production could pose the risk for severe side effects such as cytokine release syndrome (CRS) or immune effector cell-associated neurotoxicity syndrome (ICANS) (247).

TCR-like chimeric receptors might represent a major advance in T cell-based therapies by leveraging the complete TCR complex to enhance proximal signaling and anti-tumor efficacy. Constructs such as TRuCs, AbTCRs, STARs, and HITs have demonstrated superior performance in preclinical models, particularly in targeting tumors with low antigen expression and addressing limitations of second-generation ζCARs. Their ability to persist longer, resist exhaustion, and infiltrate solid tumors underscores their potential for treating refractory cancers.

### Early clinical evidence and bottlenecks of next-generation designs

Despite these promising preclinical data, clinical experience with most of these next-generation designs remains scarce: only a small number of early-phase trials currently evaluate 1XX CARs (NCT05757700), CD3ϵ-based CARs (NCT06373081), or TCR-like receptors such as TRuCs (NCT03907852), and STARs (NCT06321289, NCT03953599, NCT05548088, NCT05518357, NCT04508842) so their behavior in real-world settings is still largely unknown. In the clinic, mesothelin-targeting TRuC-T cells (NCT03907852 (242),) have shown objective responses in heavily pretreated solid tumors but also highlighted on-target/off-tumor and cytokine-mediated toxicities, illustrating both the promise and risks of engaging the full TCR-CD3 complex. Early CD19-targeting 1XX CAR T and STAR-T trials will similarly test whether tuning ζ ITAM content or harnessing complete TCR signaling can improve persistence and efficacy without exacerbating CRS, ICANS or antigen escape, but mature data are not yet available. Preclinical and first-in-human results therefore suggest that some constructs may mitigate challenges of conventional CAR T cells, yet broad implementation may be limited by manufacturing and regulatory complexity and by unresolved questions about long-term persistence, exhaustion and safety in heterogeneous tumors. Systematic head-to-head clinical evaluation of ζ-based CARs, CD3ϵ/δ/γ-containing CARs, bypassCARs and TCR-like receptors will be essential to define which designs offer the most favorable balance between efficacy, safety and feasibility, advancing the next generation of cancer immunotherapies.

## Modulation of T-cell activation

Targeting early TCR signaling events presents a promising therapeutic strategy for precisely modulating T-cell activation in autoimmune diseases, graft-*versus*-host disease (GVHD), and adoptive T-cell therapies. Because proximal TCR signaling is specific to particular T-cell populations (e.g., αβ T cells *versus* γδ T cells, or even distinct γδ subsets), this approach enables selective modulation of the desired T-cell population without inducing broad immunosuppression. Thus, targeting proximal TCR signaling could reduce infection risks and improve the patients’ quality of life.

One interesting target is the interaction between the PRS motif of CD3ϵ and the adaptor protein NCK, which is crucial for T-cell activation in response to weak antigens (48, 248). The small molecule inhibitor AX-024 specifically disrupts this interaction, showing efficacy in preclinical models of psoriasis, asthma, and multiple sclerosis by selectively reducing T-cell activation without compromising responses to strong, pathogen-derived antigens (9). A new generation of this compound is currently undergoing phase 1 and 2 clinical trials for psoriasis, atopic dermatitis and other autoimmune diseases (249), underscoring its clinical relevance (**Table 3**).

**Table 3:** Emerging therapeutic strategies targeting proximal TCR signaling.

Target	Mechanism of action	Agents	Selective effect	Disease
SH3.1 domain of NCK	Disrupts SH3.1(NCK) binding to the PRS motif of CD3ϵ	AX-024; next-generation derivates	Reduces weak-antigen activation; preserves pathogen responses	Psoriasis, atopic dermatitis (Phase 1/2); multiple sclerosis (*in vivo*)
Unique domain (UD) of LCK	Blocks UD(LCK) interaction with CD4/CD8 co-receptors	–	Preferentially would impair low-affinity autoreactive T cells	Autoimmune diseases (conceptual)
SH3 domain of LCK	Reduces SH3(LCK) binding to the RK motif of CD3ϵ	C10	Fine-tunes TCR and CD3ϵ-based CAR signaling; attenuates activation without full suppression	GVHD; reversible modulation of ϵCAR activity; restoration of exhausted ϵCARs (*in vitro*)
Kinase domain (KD) of tyrosine kinases	Inhibits the enzymatic activity of conserved KDs	Dasatinib; UNC10225387B, UNC10225263A, UNC10112761A	Reversibly blocks receptor phosphorylation; potential off-target effects	CAR-T therapy: CRS mitigation and functional restoration (*in vivo*)

Summary of pharmacologic and conceptual approaches that modulate early TCR signaling by disrupting key interactions within the CD3ϵ-NCK/LCK axis (9, 10) or inhibiting phosphorylation (11–13).

Another intervention point involves modulating LCK recruitment to the TCR. Co-receptor-associated LCK plays distinct roles in responding to high- *versus* low-affinity antigens (137) presenting a potential therapeutic window for autoimmune diseases. By targeting the interaction of the co-receptors with the unique domain of LCK, it might be possible to reduce LCK availability at the TCR, selectively impairing autoimmune T cells with low antigen affinity, while preserving high-affinity immune responses essential for pathogen defense (**Table 3**).

Alternatively, targeting the SH3 domain of LCK and its interaction with the RK motif of CD3ϵ provides another level of control. Mutating the RK motif has been shown to reduce TCR signaling, without fully abolishing it (2), making this strategy particularly appealing for conditions like GVHD, where dampening but not fully suppressing donor T-cell function is desirable. We have very recently identified a first-in-class small-molecule modulator targeting the SH3 domain of LCK (10). By reducing LCK’s interaction with CD3ϵ, this compound (named C10) attenuated allogeneic T-cell activation in an *in vitro* co-culture model, indicating potential utility in GVHD. In addition, C10 modulated the activity of CD3ϵ-containing CARs and TRuCs, lowering cytokine secretion and promoting a central-memory-like phenotype, which was associated with an improved anti-tumor response in an *in vitro* re-challenge assay (**Table 3**) (10).

By refining the signal transduction of these chimeric receptors, it may be possible to prevent adverse effects such as cytokine release syndrome (CRS) and T-cell exhaustion, while maintaining potent anti-tumor activity. Kinase inhibitors, such as dasatinib, have already demonstrated the ability to regulate CAR activity. Dasatinib inhibits ζ phosphorylation in 28ζ and BBζ CARs, reducing CRS development *in vivo*. Importantly, this inhibitory effect is reversible, allowing dasatinib to function as an “on/off switch” for CAR T cells without compromising their viability (250). Additionally, kinase inhibitors (UNC10225387B, UNC10225263A and UNC10112761A) have been shown to revive exhausted CAR T cells, restoring their anti-tumor functionality (**Table 3**) (12, 13). Inhibitors that target the initial phosphorylation steps of the CAR could achieve the same benefits while providing a more selective approach, potentially avoiding off-target effects of broad tyrosine kinase inhibitors.

Together, these strategies highlight the potential of targeted TCR modulation to address both autoimmune diseases and cancer immunotherapy, providing new avenues for precision medicine.

## Conclusion

Proximal TCR signaling, orchestrated by the interactions of the CD3 motifs with key signaling molecules such as the kinase LCK, is fundamental for T-cell activation and adaptive immune responses. Among the CD3 subunits, the CD3ϵ tail stands out as a master regulator, harboring four critical motifs – BRS, PRS, RK, and ITAM. These motifs coordinate membrane interactions, recruit essential proteins such as NCK and LCK, and facilitate ITAM phosphorylation. In contrast, the ζ chain seems to function as a signal amplifier, providing high-affinity docking sites for ZAP70 to propagate downstream signaling. CD3δ and CD3γ are less understood but appear to play regulatory roles, such as recruiting the negative regulator SHP1 and mediating TCR downregulation.

The interplay between free and co-receptor-associated pools of LCK further modulates TCR signaling dynamics. CD4-associated LCK predominantly supports helper T-cell function and stabilizes CD4, whereas CD8-associated LCK fine-tunes cytotoxic T-cell responses to weak antigens and sustains TCR-pMHC interactions during signal amplification. Co-receptors, which are not engaged by the agonist pMHC, reduce the pool of free LCK and thereby may reduce the signaling threshold.

Despite significant advancements in understanding αβ TCR signaling, γδ TCR signaling remains poorly understood. Current evidence suggests that different γδ T-cell subsets employ distinct kinases, including LCK and BLK, for signal initiation. Downstream signaling involves ZAP70 and/or SYK, but the mechanisms of kinase recruitment and the contributions of specific CD3 motifs in γδ T cells are unclear. Bridging this knowledge gap is essential for unlocking the therapeutic potential of γδ T cells, particularly in the context of tumor immunity, where their unique antigen-recognition properties could provide significant advantages.

Recent advances in CAR T-cell therapy highlight the potential of exploiting CD3 motifs to enhance therapeutic outcomes by improving anti-tumor efficacy and reducing exhaustion. Incorporating the cytoplasmic tails of CD3ϵ, CD3γ, and CD3δ into CAR constructs has been shown to improve anti-tumor responses, while lowering cytokine secretion and early exhaustion compared to second-generation ζCARs, challenging the current gold-standard of ζ-based CAR T-cell therapy. Additionally, TCR-like chimeric receptors that leverage all CD3 motifs offer a promising approach to fine-tune T-cell responses for greater efficacy and persistence in cancer immunotherapy. Now studies comparing these new chimeric receptors among each other are necessary to further advance the flied of cancer immunotherapy.

Furthermore, targeting proximal TCR signaling components presents an upcoming strategy for therapeutic intervention. Modulating the recruitment of proximal signaling molecules, such as NCK and LCK, with novel small molecules could refine the activity of endogenous TCRs in autoimmune diseases and immunopathologies or improve the function of engineered receptors in cancer therapy, while overcoming off-target effects of classical kinase inhibitors, targeting conserved enzymatic activity rather than a specific protein-protein interaction. These approaches highlight the therapeutic versatility of proximal TCR signaling modulation.

Moving forward, advancing our understanding of TCR signal initiation and its regulatory networks is essential for driving the next generation of immunotherapies. By unraveling the distinct contributions of CD3 motifs, distinct LCK pools, and the unique proximal signaling pathways in both αβ and γδ T cells, we can unlock new therapeutic opportunities. These insights will be instrumental in developing tailored, next-generation treatments that maximize therapeutic efficacy while minimizing adverse effects.
